# Silymarin as a Therapeutic Agent for Hepatocellular Carcinoma: A Multi-Approach Computational Study

**DOI:** 10.3390/metabo15010053

**Published:** 2025-01-15

**Authors:** Ouided Benslama, Sabrina Lekmine, Hamza Moussa, Hichem Tahraoui, Mohammad Shamsul Ola, Jie Zhang, Abdeltif Amrane

**Affiliations:** 1 Laboratory of Natural Substances, Biomolecules and Biotechnological Applications, Department of Natural and Life Sciences, Larbi Ben M’Hidi University, Oum El Bouaghi 04000, Algeria; 2 Biotechnology, Water, Environment and Health Laboratory, Abbes Laghrour University, Khenchela 40000, Algeria; 3 Laboratoire de Gestion et Valorisation des Ressources Naturelles et Assurance Qualité (LGVRNAQ), Faculté des Sciences de la Nature et de la Vie et des Sciences de la Terre, Université de Bouira, Bouira 10000, Algeria; 4 Département des Sciences Biologiques, Faculté des Sciences de la Nature et de la Vie et des Sciences de la Terre, Université de Bouira, Bouira 10000, Algeria; 5 Laboratoire de Génie des Procédés Chimiques, Département de Génie des Procédés, Faculté de Technologie, Université Ferhat Abbas, Sétif-1, Sétif 19000, Algeria; 6 Laboratory of Biomaterials and Transport Phenomena (LBMTP), University Yahia Fares, Médéa 26000, Algeria; 7 Ecole Nationale Supérieure de Chimie de Rennes, University of Rennes, CNRS, ISCR—UMR6226, 35000 Rennes, France; 8 Department of Biochemistry, College of Science, King Saud University, Riyadh 11451, Saudi Arabia; 9 School of Engineering, Merz Court, Newcastle University, Newcastle upon Tyne NE1 7RU, UK

**Keywords:** silymarin, hepatocellular carcinoma, network pharmacology, molecular docking, molecular dynamics simulations

## Abstract

Background: Hepatocellular carcinoma (HCC) is a prevalent and lethal form of liver cancer with limited treatment options. Silymarin, a flavonoid complex derived from milk thistle, has shown promise in liver disease treatment due to its antioxidant, anti-inflammatory, and anticancer properties. This study aims to explore the therapeutic potential of silymarin in HCC through a comprehensive in silico approach. Methods: This study employed a network pharmacology approach to identify key molecular targets of silymarin in HCC. The Genecards and Metascape databases were used for target identification and functional annotation. Molecular docking analysis was conducted on the primary silymarin components against VEGFA and SRC proteins, which are critical in HCC progression. MD simulations followed to assess the stability and interactions of the docked complexes. Results: Network pharmacology analysis identified several key molecular targets and pathways implicated in HCC. The molecular docking results revealed strong binding affinities of silymarin components to VEGFA and SRC, with Silybin A and Isosilybin B showing the highest affinities. MD simulations confirmed the stability of these interactions, indicating potential inhibitory effects on HCC progression. Conclusions: This study provides a comprehensive in silico evaluation of silymarin’s therapeutic potential in HCC. The findings suggest that silymarin, particularly its components Silybin A and Isosilybin B, may effectively target VEGFA and SRC proteins, offering a promising avenue for HCC treatment. Further experimental validation is warranted to confirm these findings and facilitate the development of silymarin-based therapeutics for HCC.

## 1. Introduction

Hepatocellular carcinoma (HCC), the most prevalent form of primary liver cancer, represents nearly 75% of liver cancer diagnoses [1]. It originates from hepatocytes, the primary functional cells of the liver [2,3]. The global incidence of HCC varies significantly, with a higher prevalence in regions with endemic HBV and HCV infections, such as East Asia and sub-Saharan Africa [4]. Clinically, HCC is often asymptomatic in its early stages, leading to late diagnosis and poor prognosis. Common symptoms in advanced stages include abdominal pain, weight loss, jaundice, and hepatomegaly [5]. Treatment strategies for HCC vary based on the disease stage and liver function, encompassing liver transplantation, surgical resection, and locoregional therapies [6]. Despite advancements in therapeutic strategies, the prognosis for HCC remains grim, with a high recurrence rate and limited survival time. Consequently, there is an urgent need for novel therapeutic approaches and preventive strategies to combat this aggressive malignancy effectively [7].

Silymarin, a complex mixture of flavonolignans derived from the seeds of the milk thistle plant (*Silybum marianum*), has garnered significant attention for its therapeutic potential, particularly in liver diseases [8]. The primary active components of silymarin include silybin, isosilybin, silydianin, and silychristin, with silybin being the most potent and extensively studied [9]. Silymarin exhibits a variety of pharmacological properties, such as antioxidant, anti-inflammatory, and antifibrotic activities [10]. These properties are largely attributed to its ability to scavenge free radicals, modulate immune responses, and inhibit the production of pro-inflammatory cytokines [11]. Furthermore, silymarin has been shown to enhance protein synthesis and promote liver regeneration, making it a promising agent in the treatment of liver disorders [12]. Preclinical studies have demonstrated that silymarin can protect hepatocytes from toxins and oxidative stress, and clinical trials have indicated its potential in managing conditions such as hepatitis, cirrhosis, and non-alcoholic fatty liver disease [13]. The multifaceted actions of silymarin on various cellular pathways underscore its potential as a therapeutic agent in hepatocellular carcinoma, as it may inhibit tumor growth, induce apoptosis, and reduce angiogenesis in liver cancer cells [14].

Given the significant potential of silymarin components in managing liver diseases and their promising pharmacological profile, this study aimed to elucidate their specific molecular targets and relevance in hepatocellular carcinoma (HCC). To achieve this, we primarily employed network pharmacology to predict and analyze the molecular targets of the silymarin components. This approach involved identifying key gene targets associated with HCC through comprehensive database searches and constructing protein–protein interaction networks. Subsequently, we used molecular docking simulations to explore the binding interactions between the silymarin components and these identified targets. To further validate and refine these interactions, we performed molecular dynamics (MD) simulations to assess the stability and dynamics of the silymarin target complexes over time. By integrating these methods, we sought to uncover the molecular interactions of silymarin components that may contribute to their therapeutic effects in HCC. This work aimed to enhance our understanding of how these components influence critical biological pathways involved in HCC, potentially guiding the development of targeted therapeutic strategies and improving current treatment options.

## 2. Materials and Methods

The molecular structure of silymarin, a complex mixture comprising silybin, isosilybin, silychristin, and silydianin, was represented as a SMILES notation and retrieved from the PubChem database (https://pubchem.ncbi.nlm.nih.gov/, accessed on 20 October 2024) [15]. This comprehensive SMILES notation, encompassing the various bioactive components of silymarin, was then used to query the SwissTargetPrediction database (http://www.swisstargetprediction.ch/, accessed on 20 October 2024) [16]. The prediction aimed to identify the most likely molecular targets associated with silymarin and its individual components.

### 2.1. Identification of Targets Associated with HCC

To identify targets associated with hepatocellular carcinoma (HCC), we accessed the Genecards and OMIM databases. The Genecards database [17], known for its comprehensive and user-friendly information on human genes, was utilized to gather data relevant to hepatocellular carcinoma (https://www.genecards.org/, accessed on 20 October 2024). Additionally, the OMIM database [18], which provides detailed information on genetic and hereditary diseases, was consulted (https://www.omim.org/, accessed on 20 October 2024). By searching for hepatocellular carcinoma in both databases, we identified key targets related to this condition. Targets identified from Genecards and OMIM (non-redundant) were merged, and a Venn diagram was created to illustrate the genes common to both silymarin and HCC targets.

### 2.2. Protein–Protein Interaction (PPI) Network Construction

The common gene targets were uploaded to the STRING database [19] (https://string-db.org/, accessed on 20 October 2024) for analysis. The species was set to *Homo sapiens*, and the resulting interaction network was then imported into Cytoscape 3.10.0. Statistical analysis was subsequently conducted based on degree values. The CytoHubba plugin [20] was employed to identify the top 10 hub genes using the degree method. Finally, a network illustrating the connections between silymarin, targets, and pathways associated with hepatocellular carcinoma was generated.

### 2.3. Gene Ontology (GO) and Pathway (KEGG) Enrichment Analysis of Potential Targets

We conducted Gene Ontology (GO) functional annotation to categorize genes based on their biological roles, leveraging sequence similarity, experimental evidence, and the available literature [21]. Additionally, the Kyoto Encyclopedia of Genes and Genomes (KEGG) pathway analysis was employed to map out the complex interactions between genes, proteins, and small molecules within various biological contexts [22], For our study, we utilized the ShinyGo database v 0.80 (http://bioinformatics.sdstate.edu/go74/, accessed on 22 October 2024) to conduct both GO functional annotation and KEGG pathway enrichment analyses [23].

### 2.4. Molecular Docking

The molecular docking simulations were conducted to investigate the binding interactions between silymarin and the target proteins, selected based on the results obtained from the network pharmacology analysis. Initially, the three-dimensional structures of the target proteins were retrieved from the Protein Data Bank (PDB). The protein structures were then prepared using the Molecular Operating Environment (MOE 2015.10) software [24], which involved adding hydrogen atoms, removing water molecules, and optimizing the protein structure. The structures of silymarin and reference ligands were obtained from the PubChem database. To ensure compatibility with the docking software, ligand structures were subjected to energy minimization and conversion into the appropriate file format using MOE.

The active sites of the target proteins were identified using the CASTp 3.0 server (http://sts.bioe.uic.edu/castp) [25] and the active site search tool within MOE. Molecular docking was carried out using MOE, employing a semiflexible docking approach. The docking parameters were set to ensure a thorough exploration of possible binding conformations.

### 2.5. Molecular Dynamics (MD) Simulation

The initial structures of the protein–ligand complexes were obtained from the molecular docking results, and the protein and ligand structures were prepared using MOE software to ensure proper protonation states and the removal of any unnecessary crystallographic water molecules. Force field parameters were assigned using the AMBER99SB force field for the proteins and the General Amber Force Field (GAFF) for the ligands. The complexes were solvated in a TIP3P water box with a 10 Å buffer, and appropriate counterions were added. Energy minimization was performed to remove any steric clashes under an NVT ensemble. Equilibration was carried out for 500 ps under an NPT ensemble to ensure stable pressure and temperature conditions. Production MD simulations were then run for 100 ns with a time step of 2 fs, maintaining a constant temperature of 300 K using the Langevin thermostat and a pressure of 1 atm using the Berendsen barostat [26,27]. GROMACS 2024.4 software was employed for all MD simulations [28]. Trajectory analysis was conducted using the CPPTRAJ module in AMBER.

### 2.6. MM-PBSA Analysis

To further analyze the binding affinities of the ligands, we applied the Molecular Mechanics/Poisson–Boltzmann Surface Area (MM/PBSA) methodology. We utilized the g_mmpbsa package to perform the MM/PBSA calculations, which included evaluating various energy components. The total binding free energy was determined by summing these energy contributions. This procedure follows the protocols outlined by Kumari et al. (2014) [29].

**Figure 1:**
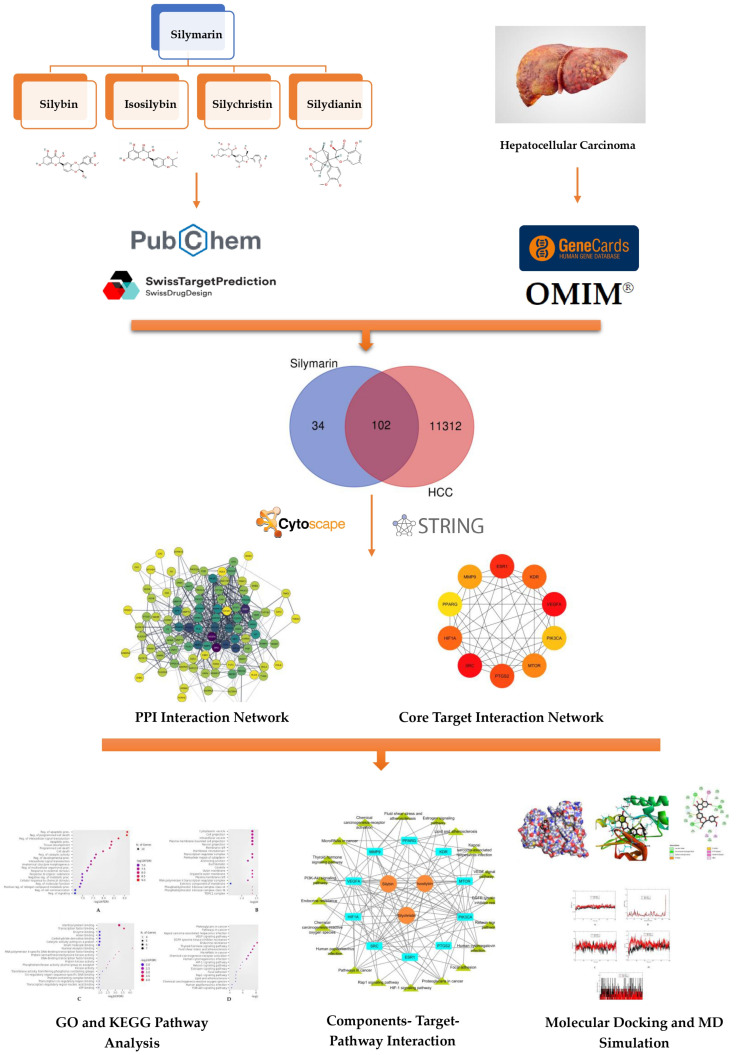
Overview of the research workflow.

Figure 1 presents a graphical representation of this study’s workflow.

## 3. Results

### 3.1. Identification of Potential Targets of Silymarin and Hepatocellular Carcinoma

A total of 136 potential target genes were identified for silymarin using the Swiss Target Prediction tool (http://www.swisstargetprediction.ch/). Notably, among the components of silymarin, only silybin, isosilybin, and silychristin showed identifiable targets, whereas silydianin did not reveal any known targets. In parallel, we compiled a total of 11,414 targets related to hepatocellular carcinoma (HCC) by aggregating data from the Genecards and OMIM databases. To visualize the overlap, Venn diagrams were created. This analysis revealed 102 targets that are common to both silymarin and HCC, highlighting their potential intersection.

### 3.2. Analysis of PPI Interaction and Core Target Networks

**Figure 2:**
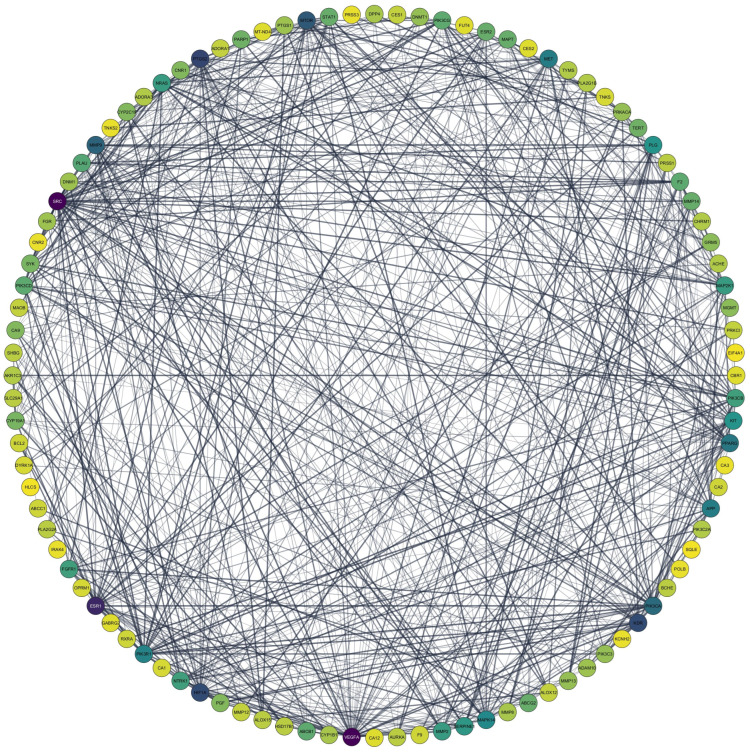
PPI network of 102 coincident targets of HC and silymarin.

**Figure 3:**
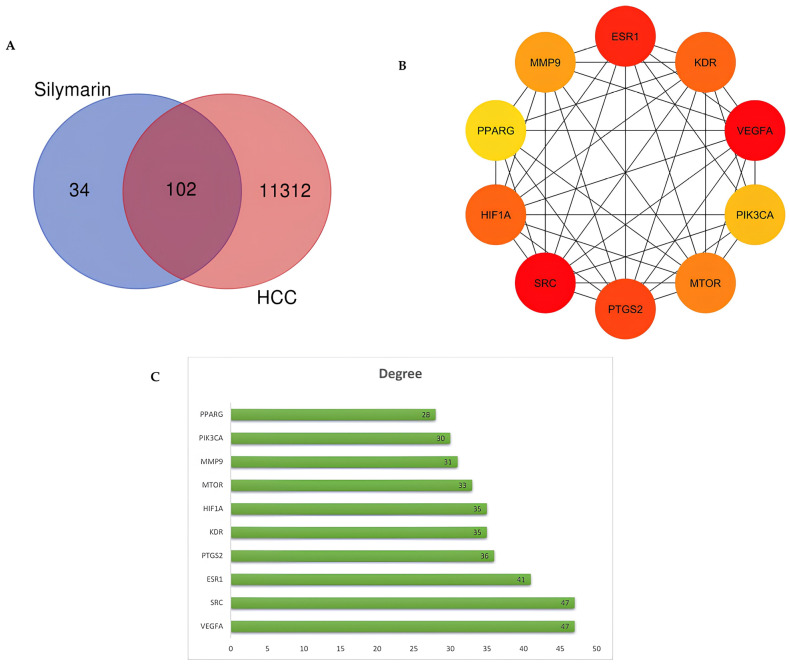
(**A**) Venn diagram shows the targets of silymarin and HCC. (**B**) The network of top 10 core HCC-related targets screened using degree values. (**C**) Ten core targets ranked by degree values.

The PPI network of the common genes results is illustrated in Figure 2. The network comprised 102 nodes and 647 edges with an average centrality degree of the neighbors of 12,947, highlighting its complexity. The interaction degree of the targets is depicted by varying shades of color, as shown in Figure 2. Additionally, Figure 3B,C present the top 10 targets with the highest degree values, with vascular endothelial growth factor A (VEGFA) and tyrosine–protein kinase SRC (SRC) emerging as the targets with the highest degree value of 47.

### 3.3. GO Functional Annotation and KEGG Enrichment of Core Targets

**Figure 4:**
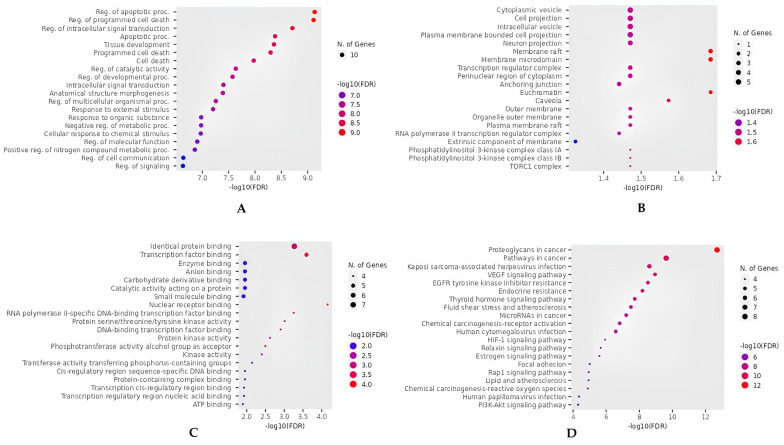
Functional annotation and KEGG pathway enrichment analysis of 10 core gene targets. (**A**) Dotplot chart showing the top 20 BPs. (**B**) Dotplot chart showing the top 20 CCs. (**C**) Dotplot chart showing the top 20 MFs. (**D**) Dotplot chart showing the top 20 KEGG pathways.

For a detailed understanding of the key mechanisms involved in the action of silymarin against hepatocellular carcinoma, we conducted GO functional and KEGG enrichment annotation analyses for an in-depth gene annotation. Based on biological process (BP) results, the main upregulated processes include the apoptotic process, programmed cell death, intracellular signal transduction, tissue development, catalytic activity, and other related processes (Figure 4A). Analysis of cellular components (CCs) shows that most core genes are included in cytoplasmic vesicles, cell projections, intracellular vesicles, plasma membrane-bounded cell projections, etc. (Figure 4B). Molecular function (MF) analysis indicates that the genes are involved in activities such as identical protein binding, transcription factor binding, enzyme binding, anion binding, carbohydrate derivative binding, and more (Figure 4C). Moreover, the data from KEGG pathway analysis reveal that silymarin exerts its therapeutic effects primarily by modulating the proteoglycans in cancer, Kaposi sarcoma-associated herpesvirus infection, the VEGF signaling pathway, EGFR tyrosine kinase inhibitor resistance, endocrine resistance, the thyroid hormone signaling pathway, and additional pathways, thereby offering a multi-target approach to the treatment of the hepatocellular carcinoma (Figure 4D).

**Figure 5:**
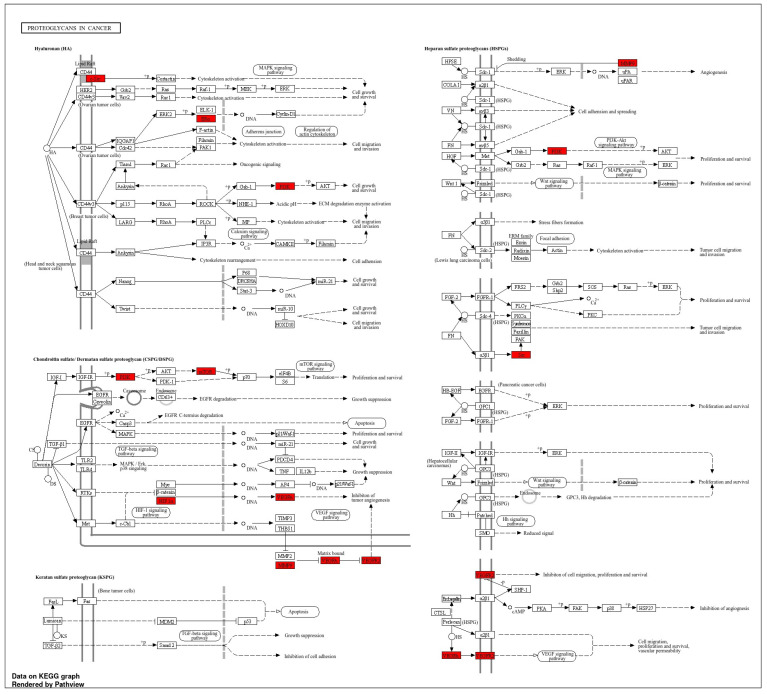
Diagram of pathways involving potential targets identified through KEGG analysis. The red sections highlight the silymarin targets associated with HC.

Given these data and the combination of key targets, the proteoglycans in the cancer pathway, identified as the main pathway by KEGG analysis, were further explored using KEGG via Pathview (Figure 5). A visual pathway map was created to illustrate the mechanism of silymarin in the treatment of hepatocellular carcinoma, highlighting its primary effects on angiogenesis, cell proliferation and survival, and tumor cell migration and invasion. In the context of HCC, silymarin exerted both direct and indirect regulatory effects on the condition.

### 3.4. Silymarin–Target–Pathway Network

**Figure 6:**
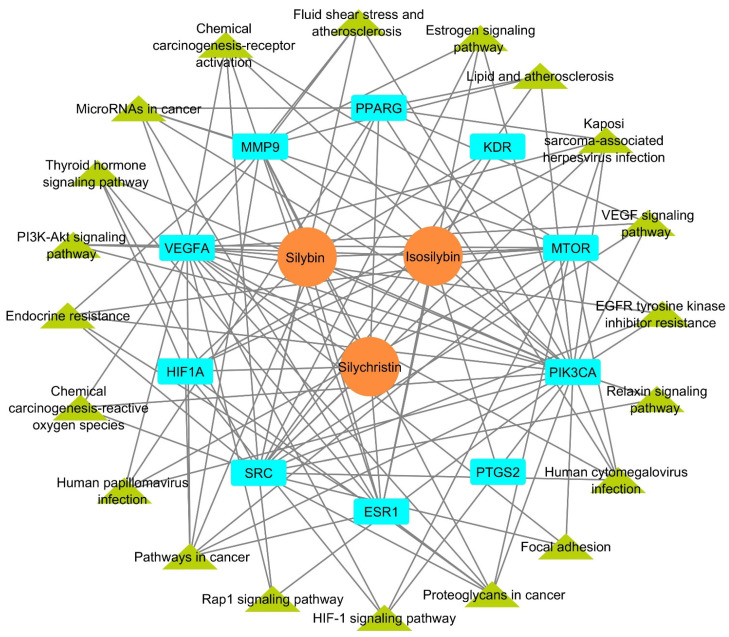
Silymarin–target–pathway network. The orange circles represent the three active components of silymarin, the cyan rectangles represent the core genes, and the green triangles represent the pathways linked to the core genes.

To explore the multi-target effects of silymarin in HCC, two separate networks were constructed: the compound–target network and the target-pathway network. These two networks were subsequently merged to form an integrated compound–target-pathway network, resulting in a structure with 33 nodes and 112 edges (Figure 6).

### 3.5. Molecular Docking

Among the 10 hub targets identified in our network pharmacology analysis, VEGFA and SRC exhibited the highest degree of centrality, with degree values of 47, and were closely associated with the pathways identified in the KEGG analysis. Consequently, we obtained the three-dimensional structure of VEGFA (PDB ID: 1VPF) and SRC (PDB ID: 1U5E) from the Protein Data Bank to use as the protein receptors in our docking simulations. The structures of silymarin’s active components were retrieved from PubChem. Molecular docking with these components was performed using MOE software, employing a semiflexible docking approach.

In the case of VEGFA, 1,2,3,4,6-penta-O-galloyl-beta-D-glucose (PGG) was used as the reference for comparison due to its reported direct inhibitory effects on VEGFA [30]. The investigation of the active site of VEGFA was conducted using the CASTp program and the active site search tool in MOE software. Both methods consistently identified the same geometric localization of the binding pocket. The identified site comprises key residues, including Gly58, Gly59, Cys60, Cys61, Asn62, Asp63, Glu64, Leu66, Glu67, Cys68, and Lys107 from chain 1 and Leu32, Asp34, Phe36, Tyr45, Ile46, Phe47, Lys48, Ser50, and Cys51 from chain 2. This consensus between CASTp and MOE confirms the reliability of the docking results and the significance of these residues in the binding interactions.

**Figure 7:**
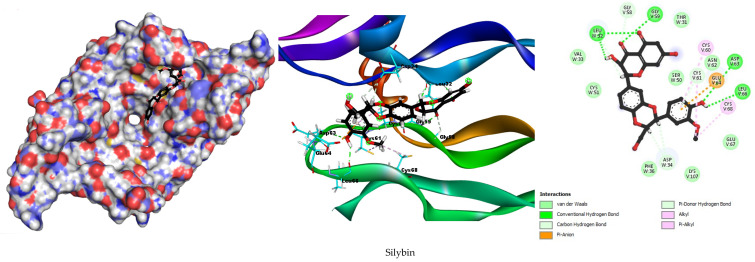
Molecular docking 2D and 3D diagrams of silybin with the highest degree hub target VEGFA (1VPF).

The results of the interaction energy, hydrogen bonding, and hydrophobic interactions are summarized in Table 1. Additionally, the geometric disposition of silymarin’s active components within the active site of VEGFA, along with the established interactions, are illustrated in Figure 7 and Supplementary Figure S1.

Among the compounds analyzed, PGG exhibited the highest binding energy of −8.0997 kcal/mol, surpassing that of the primary silymarin components. This suggests a stronger affinity for VEGFA. PGG also formed an extensive network of 10 hydrogen bonds involving key residues such as Cys68, Cys61, Asp34, Gln37, Thr31, Ser50, Val33, Gly58, and Cys57, which highlights its ability to establish stable interactions within the VEGFA binding site. Additionally, PGG demonstrated hydrophobic interactions with Pro70, Cys57, Cys61, and Cys60 and an electrostatic interaction with Glu64, further supporting its robust binding affinity and specificity.

Silybin, a major component of silymarin, displayed a competitive binding energy of −7.7234 kcal/mol, which, while slightly lower than the binding energy of PGG (−8.0997 kcal/mol), remains highly indicative of a strong binding affinity for VEGFA. Notably, silybin formed 10 hydrogen bonds with key residues, such as Cys61, Gly59, Gly58, Leu32, Asp34, Asp63, and Leu66, matching PGG in its ability to establish a robust hydrogen bonding network. Additionally, its hydrophobic contacts with Cys60 and Cys68, alongside an electrostatic interaction with Glu64, mirror the diverse interaction profile observed for PGG. These findings suggest that silybin holds comparable potential to PGG as a VEGFA inhibitor, particularly given its strong network of interactions and its position as a natural compound with therapeutic promise.

Isosilybin, another active component of silymarin, demonstrated a binding energy of −7.6397 kcal/mol, forming nine hydrogen bonds involving key residues such as Cys61, Glu64, Asn62, Gly58, Gly59, Asp63, and Ser63. The extensive hydrogen bonding network and interactions with hydrophobic residues, like Cys60, Cys68, and Phe36, along with an additional interaction with Anp34, indicate a substantial binding affinity and stability within the active site of VEGFA.

Silychristin displayed a binding energy of −6.7097 kcal/mol, forming four hydrogen bonds with residues Glu64, Gly59, and Asp34. Despite the lower number of hydrogen bonds, the involvement of key hydrophobic residues Phe36, Ile46, and Cys60, and an electrostatic interaction with Glu64, suggest a moderate but significant interaction with the VEGFA protein.

**Figure 8:**
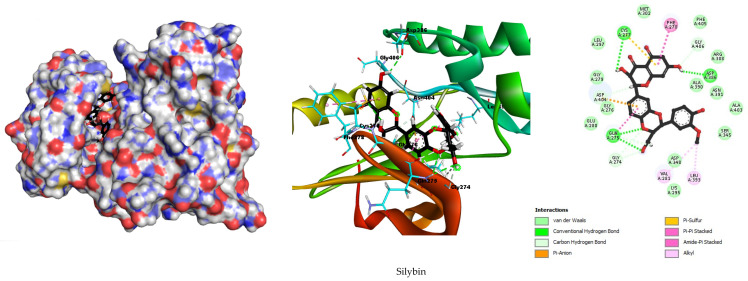
Molecular docking 2D and 3D diagrams of silybin with the highest degree hub target SRC (3U51).

The molecular docking analysis was extended to include SRC (PDB ID: 3U51), which, along with VEGFA, exhibited the highest degree of centrality in our network pharmacology analysis. The co-crystallized inhibitor MC1 was included as a reference for comparison. To ensure the reliability of our docking procedure, a re-docking of the co-crystallized ligand MC1 was performed, yielding a Root Mean Square Deviation (RMSD) value of 0.651. This RMSD value, being below the commonly accepted threshold of 2.0, confirms the accuracy and validity of our docking protocol. The results of the docking simulations are summarized in Table 2. Additionally, Figure 8 and Supplementary Figure S2 illustrate the spatial arrangement of silymarin’s active components within the SRC active site, along with the established interactions.

The co-crystallized inhibitor MC1 displayed a binding energy of −10.6949 kcal/mol and formed an extensive network of 17 hydrogen bonds. The high number of hydrogen bonds, along with hydrophobic interactions, involved Ile336, Gly279, and Cys277 and electrostatic interactions with Asp404 and Asp348.

In comparison, silybin exhibited a binding energy of −8.7466 kcal/mol and formed nine hydrogen bonds with key residues Cys277, Asp404, Gln275, Gly274, and Gly406. These hydrogen bonds, along with hydrophobic interactions involving Phe278, Gln275, Val281, and Leu393, contribute to the stability and specificity of silybin’s binding within the active site of SRC. Additionally, electrostatic interactions with residues Cys277 and Asp404 further reinforce the binding affinity of silybin.

Isosilybin showed a binding energy of −8.7382 kcal/mol and formed five hydrogen bonds with residues Ala403, Asp386, Asp404, and Glu280. The hydrophobic interactions with Val281, Gln275, and Cys277 coupled with electrostatic interactions involving Lys295, Met314, and Asp404.

Silychristin demonstrated a binding energy of −8.6096 kcal/mol, forming seven hydrogen bonds with residues Glu280, Gly279, Leu273, Gly344, and Asp348. Hydrophobic interactions were observed with Leu393, Val281, and Leu273, while electrostatic interactions involved Asp404 and Cys177.

### 3.6. Molecular Dynamics Simulations

**Figure 9:**
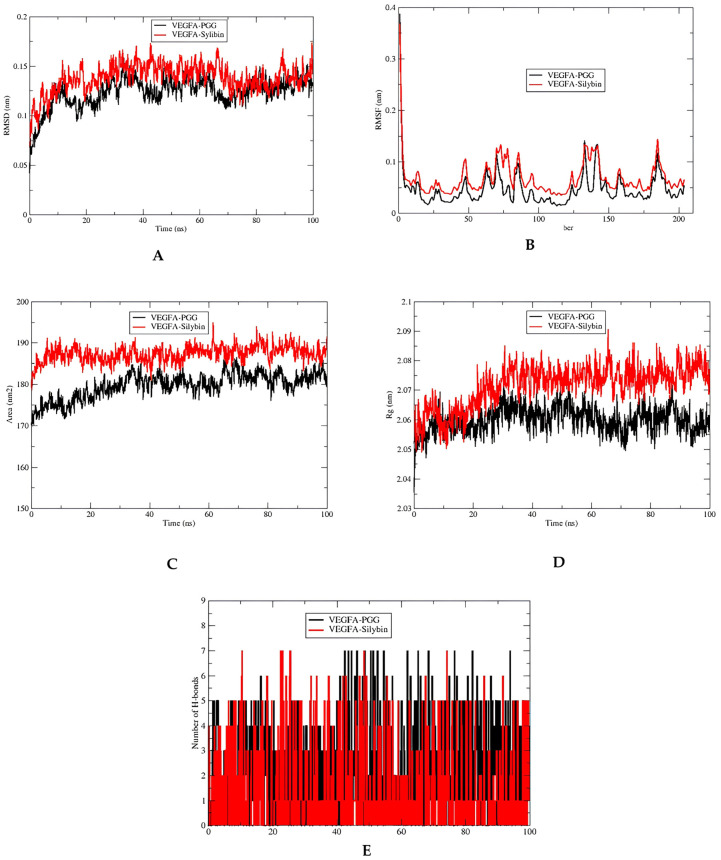
(**A**) RMSD of the VEGFA–silybin and VEGFA-PGG complexes during 100 ns MD simulation. (**B**) RMSF of the VEGFA–silybin and VEGFA-PGG complexes during 100 ns MD simulation. (**C**) SASA of the VEGFA–silybin and VEGFA-PGG complexes during 100 ns MD simulation. (**D**) Radius of gyration of the VEGFA–silybin and VEGFA-PGG complexes during 100 ns MD simulation. (**E**) Number of H-bonds of the VEGFA–silybin and VEGFA-PGG complexes during 100 ns MD simulation.

**Figure 10:**
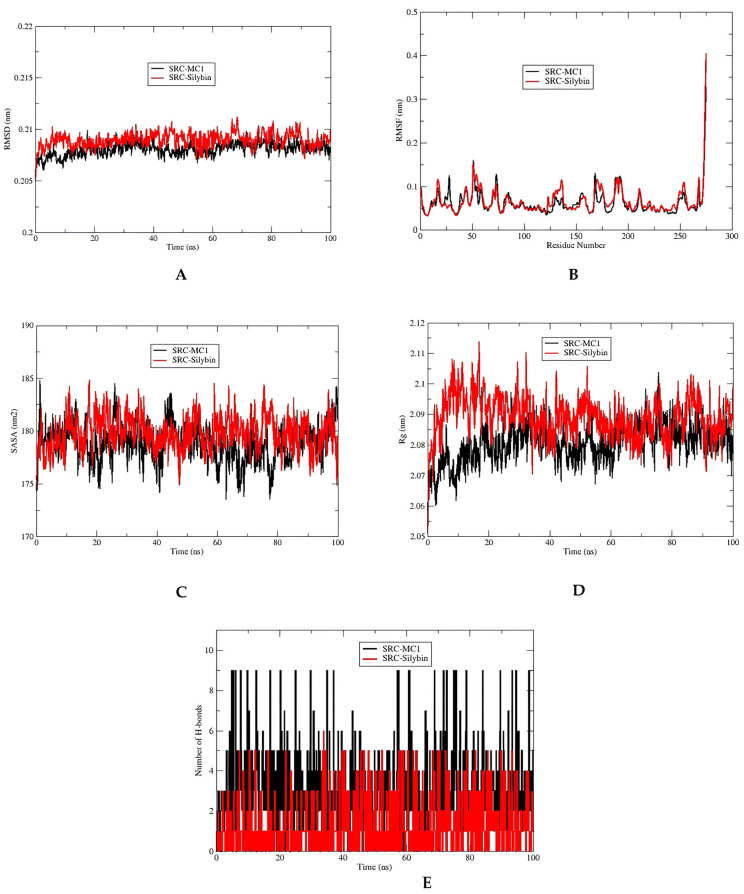
(**A**) RMSD of the SRC–silybin and SRC co-crystallized inhibitor complexes during 100 ns MD simulation. (**B**) RMSF of the SRC–silybin and SRC co-crystallized inhibitor complexes during 100 ns MD simulation. (**C**) SASA of the SRC–silybin and SRC co-crystallized inhibitor complexes during 100 ns MD simulation. (**D**) Radius of gyration of the SRC–silybin and SRC co-crystallized inhibitor complexes during 100 ns MD simulation. (**E**) Number of H-bonds of the SRC–silybin and SRC co-crystallized inhibitor complexes during 100 ns MD simulation.

After conducting the molecular docking analysis, we proceeded with molecular dynamics (MD) simulations for three complexes: VEGFA with silybin, SRC with its co-crystallized inhibitor MC1 as a reference, and SRC with silybin. Silybin was chosen for this extended study because it showed the best molecular docking results among the three silymarin components with the two best core targets: VEGFA and SRC. Additionally, silybin is known to be the most represented molecule in the silymarin complex. The MD simulation analysis is summarized in Table 3 and illustrated in Figure 9 and Figure 10.

The results of the molecular dynamics simulation for the studied complexes are summarized in Table 3. For the VEGFA-PGG complex, the average RMSD was 0.12 ± 0.01 nm, the RMSF was 0.05 ± 0.04 nm, the Rg was 2.05 ± 0.02 nm, the SASA was 179.42 ± 3.07 nm^2^, and the maximum number of hydrogen bonds was 7. For the VEGFA–silybin complex, the average RMSD was 0.14 ± 0.01 nm, the RMSF was 0.07 ± 0.03 nm, the Rg was 2.07 ± 0.07 nm, the SASA was 187.85 ± 3.10 nm^2^, and the maximum number of hydrogen bonds was also 7. Comparing these results, the VEGFA-PGG complex demonstrated a slightly lower RMSD compared to the VEGFA–silybin complex, suggesting marginally higher stability. The RMSF for VEGFA-PGG was also lower, indicating less flexibility within this complex. The Rg values were close, with VEGFA-PGG showing a slightly more compact conformation. The SASA for VEGFA-PGG was slightly lower than that of VEGFA–silybin, suggesting marginally reduced solvent exposure. Both complexes exhibited an equal maximum number of hydrogen bonds (7), highlighting their comparable ability to establish strong and stable interactions with the VEGFA binding site. These findings suggest that while VEGFA–silybin demonstrates good binding characteristics, VEGFA-PGG may offer a slightly more stable and compact binding profile, supporting its potential as a strong VEGFA inhibitor.

Regarding the SRC-MC1 complex, the average RMSD was 0.20 ± 0.01 nm, the RMSF was 0.06 ± 0.03 nm, the Rg was 2.08 ± 0.06 nm, the SASA was 178.86 ± 1.80 nm^2^, and the maximum number of hydrogen bonds was 6. For the SRC–silybin complex, the average RMSD was 0.21 ± 0.01 nm, the RMSF was 0.07 ± 0.03 nm, the Rg was 2.10 ± 0.07 nm, the SASA was 179.82 ± 1.66 nm^2^, and the maximum number of hydrogen bonds was 9. Comparing these results, the SRC–silybin complex showed slightly higher RMSD values than the SRC-MC1 complex, indicating slightly more backbone movement. The RMSF for SRC–silybin was also higher, suggesting increased flexibility. The Rg values indicated that SRC–silybin had a less compact structure compared to SRC-MC1. The SASA values were similar, with SRC–silybin having a slightly higher value. The SRC–silybin complex formed more hydrogen bonds compared to the SRC-MC1 complex.

### 3.7. MMPBSA Analysis

The results of the MMPBSA analysis, summarized in Table 4, provide a comprehensive evaluation of the binding interactions and energetics for the VEGFA and SRC protein–ligand complexes.

For the VEGFA-PGG complex, the total binding energy is −96.26 ± 11.32 kJ mol^−1^, indicating a stronger interaction compared to the VEGFA–silybin complex. The van der Waals energy contributes the most significantly to the binding energy, with a value of −102.16 ± 9.05 kJ mol^−1^, emphasizing the importance of hydrophobic interactions in stabilizing this complex. The electrostatic energy, though relatively lower at −4.25 ± 2.05 kJ mol^−1^, still supports the binding. The polar solvation energy is 19.36 ± 8.57 kJ mol^−1^, indicating a desolvation penalty, which is effectively mitigated by the SASA energy of −9.21 ± 2.18 kJ mol^−1^, reflecting efficient burial of hydrophobic regions. In contrast, the VEGFA–silybin complex shows a less favorable total binding energy of −77.41 ± 12.95 kJ mol^−1^, suggesting weaker binding. The van der Waals energy is −89.47 ± 11.25 kJ mol^−1^, which is lower compared to PGG, indicating reduced hydrophobic interactions. The electrostatic energy is comparable at −5.02 ± 0.35 kJ mol^−1^, while the polar solvation energy is slightly less favorable at 22.18 ± 13.15 kJ mol^−1^, further contributing to the weaker binding. Additionally, the SASA energy of −5.10 ± 0.42 kJ mol^−1^ suggests less efficient burial of hydrophobic regions compared to PGG.

For the SRC-MC1 complex, the total binding energy is −184.75 ± 13.70 kJ mol^−1^, indicating a very strong binding interaction. The van der Waals energy is the most significant contributor at −200.78 ± 7.95 kJ mol^−1^, underscoring the dominant role of hydrophobic interactions in stabilizing this complex. The electrostatic energy is also favorable at −17.55 ± 1.92 kJ mol^−1^, enhancing the interaction further. However, the polar solvation energy contributes a penalty of 45.60 ± 6.75 kJ mol^−1^, which is partially offset by the favorable SASA energy of −12.02 ± 0.65 kJ mol^−1^, reflecting efficient burial of hydrophobic surfaces. In comparison, the SRC–silybin complex exhibits a less favorable total binding energy of −143.62 ± 12.88 kJ mol^−1^, suggesting weaker binding compared to MC1. The van der Waals energy is −148.32 ± 10.68 kJ mol^−1^, which is lower than that of MC1, indicating reduced hydrophobic stabilization. Interestingly, the electrostatic energy is slightly higher in magnitude at −22.05 ± 2.65 kJ mol^−1^, suggesting that electrostatic interactions play a more prominent role in this complex. The polar solvation energy is 38.50 ± 8.12 kJ mol^−1^, reflecting a slightly lower desolvation penalty compared to MC1. However, the SASA energy is comparable at −11.75 ± 1.59 kJ mol^−1^.

## 4. Discussion

Silymarin, traditionally used for its hepatoprotective properties, has been shown to have significant therapeutic potential in treating hepatocellular carcinoma through various studies [12,31]. However, the underlying molecular mechanisms by which silymarin exerts its effects remain incompletely understood. Thus, exploring the interactions between silymarin’s active compounds and key molecular targets can help elucidate its beneficial effects and optimize its therapeutic use. In this study, we employed network pharmacology, molecular docking, and MD simulation analyses to investigate the mechanisms by which silymarin influences HCC.

Through our network analysis, derived from the intersection of target genes associated with both HCC and silymarin, we identified 10 hub genes that play a critical role in the disease’s progression. Additionally, we performed KEGG pathway enrichment and GO functional annotation analyses to identify the main pathways and biological processes involved. These analyses revealed crucial insights into the signaling pathways and functional annotations that silymarin may influence, thereby providing a deeper understanding of its potential therapeutic mechanisms against HCC. In this study, we focused on two critical proteins that emerged as the hub targets with the highest degree values from our network pharmacology analysis, VEGFA and SRC.

Vascular endothelial growth factor A (VEGFA) plays a central role in angiogenesis, the formation of new blood vessels from existing ones. This process is essential for metastasis and tumor development, supplying nutrients and oxygen for dividing cancer cells [32]. The overexpression of VEGFA has been observed in many cancers, including HCC, and is associated with poor prognosis and increased tumor aggressiveness [33]. In HCC, VEGFA promotes the proliferation, migration, and invasion of endothelial cells, contributing to the formation of a dense network of blood vessels within the tumor. This not only supports the rapid growth of the tumor but also facilitates the spread of cancer cells to other parts of the body [34]. Therapies targeting VEGFA, such as sunitinib, have shown efficacy in inhibiting angiogenesis and controlling tumor growth in HCC patients [35]. Thus, the identification of VEGFA as a key target in our study underscores its importance in HCC progression and highlights the potential of silymarin to interfere with angiogenesis pathways.

SRC is a tyrosine kinase that operates without a receptor and plays a role in numerous cellular processes, including proliferation, survival, migration, and angiogenesis [36]. It acts as an oncogene in many cancers, including HCC, where its overactivation is correlated with metastasis and tumor progression [37]. In HCC, SRC is implicated in the activation of multiple downstream signaling pathways that promote oncogenic behaviors. For example, SRC activation leads to the phosphorylation and activation of several substrates involved in cell motility and invasion, such as focal adhesion kinase (FAK) and paxillin [38,39]. Furthermore, SRC can modulate the tumor microenvironment by promoting angiogenesis and altering immune responses, thereby facilitating tumor growth and dissemination [40]. The inhibition of SRC has been explored as a therapeutic strategy in HCC. SRC inhibitors, such as dasatinib, have shown promise in preclinical studies by reducing metastasis and tumor growth [15]. The selection of SRC as a hub gene in our network analysis suggests that silymarin’s active compounds might exert their anti-HCC effects partly through the modulation of SRC-related pathways.

Given their central roles and high degree values, we selected these proteins for detailed molecular docking studies to explore the binding affinities and interaction mechanisms of silymarin’s active components compared to known anti-VEGF and anti-SRC drugs. The insights gained from these docking simulations aim to elucidate the potential therapeutic effects of silymarin in inhibiting these critical pathways in hepatocellular carcinoma.

The molecular docking results reveal promising interactions between the active components of silymarin, especially silybin, and VEGFA. Silybin demonstrated a binding energy of −7.7234 kcal/mol, forming ten hydrogen bonds with key residues such as Cys61, Gly59, Gly58, Leu32, and Asp34. This strong binding affinity is further supported by hydrophobic interactions with Cys60 and Cys68 and an electrostatic interaction with Glu64. The extensive network of interactions suggests that silybin may have the potential to stabilize the VEGFA binding site, which could indicate its role as a possible inhibitor of VEGFA. This finding points to the potential utility of silybin in cancer therapies targeting angiogenesis; however, further experimental validation is essential to confirm these computational predictions and fully evaluate its therapeutic relevance.

Similarly, isosilybin, another significant component of silymarin, demonstrated a binding energy of −7.6397 kcal/mol, along with a robust hydrogen bonding network and interactions with hydrophobic residues such as Cys60, Cys68, and Phe36. These computational findings suggest that isosilybin may contribute to the potential therapeutic applications of silymarin in inhibiting VEGFA. These results lay the groundwork for future experimental studies to confirm and build upon the insights gained from this computational analysis.

In this study, PGG (1,2,3,4,6-penta-O-galloyl-β-D-glucose) serves as a reference VEGF-A inhibitor compound. The selection of PGG as a reference molecule is strongly supported by the promising findings reported in the study by Ren et al. (2023) [30]. In their work, PGG was ranked as one of the most effective small molecular compounds among several candidates. Notably, PGG demonstrated the best inhibitory effect on the proliferation of AGS and HGC27 cells in a concentration- and time-dependent manner. This study also revealed that PGG significantly inhibited cell cloning, migration, and invasion, while inducing apoptosis in AGS and HGC27 cells, underscoring its potent biological activity. Furthermore, the molecular dynamics simulations conducted in Ren et al.’s study showed that PGG exhibited superior binding to the VEGFA target protein compared to other small molecules. These results were consistent with the molecular docking findings, reinforcing the notion that PGG has a strong affinity for VEGFA and is a promising candidate for inhibiting angiogenesis. Based on these in silico and in vitro results, PGG serves as an ideal reference molecule for comparison, as it has demonstrated both potent biological activity and strong molecular interactions with VEGFA, making it an appropriate standard for evaluating the potential of other VEGFA inhibitors, such as the active components of silymarin.

The docking results for PGG revealed a binding energy of −8.0997 kcal/mol, showing a highly favorable interaction with VEGFA. PGG forms an extensive network of interactions, including ten hydrogen bonds with residues like Cys68, Cys61, and Asp34, along with hydrophobic interactions at Cys60, Cys57, and Pro70 and an electrostatic interaction with Glu64. The high binding affinity and extensive interaction profile observed for PGG in this study align with its promising anti-VEGF activity, as demonstrated in both in silico and experimental in vitro studies. This makes PGG a valuable reference compound for comparing the binding efficacy of other potential VEGFA inhibitors, such as silymarin’s active components.

Despite PGG showing a stronger binding affinity, silybin’s interaction with VEGFA remains noteworthy due to its comparable binding energy and the formation of a substantial network of hydrogen bonds and key interactions. These findings suggest that silybin could serve as a competitive alternative to PGG, offering the potential for VEGFA inhibition. While these results underscore the therapeutic promise of silymarin’s active components in targeting VEGFA, further experimental validation is required to confirm these computational observations and assess their practical relevance in therapeutic applications.

The molecular docking analysis for SRC complements our investigation of VEGFA, both of which exhibited high centrality in our network pharmacology analysis. The docking results, summarized in Table 2 and illustrated in Figure 6, highlight the potential of silymarin’s active components as effective SRC inhibitors. The co-crystallized inhibitor MC1, used as a benchmark, displayed a binding energy of −10.6949 kcal/mol and formed an extensive network of 17 hydrogen bonds, supplemented by significant hydrophobic and electrostatic interactions. This strong binding profile highlights the efficacy of MC1 as an SRC inhibitor.

In comparison, silybin exhibited a binding energy of −8.7466 kcal/mol, forming nine hydrogen bonds with critical residues, such as Cys277 and Asp404. The presence of hydrophobic interactions with residues like Phe278 and electrostatic interactions with Cys277 further strengthens the stability and specificity of silybin’s binding to SRC. Isosilybin demonstrated a slightly higher binding energy of −8.8382 kcal/mol and formed five hydrogen bonds with residues, including Ala403 and Asp404. Its binding is reinforced by hydrophobic interactions with Val281 and electrostatic interactions involving Lys295.

Silychristin, with a binding energy of −8.6096 kcal/mol, displayed seven hydrogen bonds with residues, such as Glu280 and Gly279. The hydrophobic interactions with Leu393 and electrostatic interactions with Asp404 contribute to its binding affinity. Although the binding energy of silychristin is slightly lower than that of silybin and isosilybin, the observed interactions suggest a meaningful and significant affinity for SRC. Our results indicate that silymarin’s active components, particularly silybin and isosilybin, exhibit substantial binding affinity to SRC, with values comparable to those of known inhibitors. These findings highlight the potential therapeutic value of silymarin in targeting SRC, suggesting that it is a promising candidate for further investigation in the treatment of hepatocellular carcinoma. However, given the computational nature of this study, these results should be considered preliminary and warrant additional experimental confirmation.

In both targets, VEGFA (1VPF) and SRC (3U51), hydrogen bonding interactions are the dominant force governing the binding of silymarin active components. The docking results clearly demonstrate that silybin, isosilybin, and silychristin form multiple hydrogen bonds with key residues on both targets, with silybin showing the highest number of hydrogen bonds in both cases. These hydrogen bonds are essential for stabilizing the ligand–target complex and significantly contribute to the overall binding affinity. While hydrophobic and electrostatic interactions also play a role in the stabilization of these complexes, they are secondary to the hydrogen bonding interactions, which appear to be the primary drivers of binding. This observation indicates that the strong hydrogen bond formation by silymarin components with critical residues on both VEGFA and SRC could contribute to their potential therapeutic activity, especially in the modulation of angiogenesis and cellular signaling pathways. These findings provide a basis for further experimental studies to validate and expand upon these computational insights.

The docking results of silybin, isosilybin, and silychristin with VEGF and SRC reveal important insights into the binding mechanisms, particularly in relation to the secondary structure elements of the proteins. Our analysis shows that these compounds predominantly interact with β-sheets and α-helices, which are crucial structural domains in both VEGF and SRC. In VEGF, key interactions occur with residues in the β-sheet regions, such as Gln275, Gly274, and Asp404, and hydrophobic interactions are observed with residues in α-helical regions, like Cys277 and Leu393. Similarly, for SRC, significant binding interactions are observed in both β-sheet regions (Glu280, Asp386) and α-helical regions (Leu273, Val281), with additional stabilizing electrostatic interactions with Asp404 and Cys177. These findings highlight the importance of both secondary structure elements in stabilizing the binding of the compounds, reinforcing their potential therapeutic efficacy.

The molecular dynamics (MD) simulation provides an in-depth understanding of the interactions and stability of the protein–ligand complexes under near-physiological conditions. In this study, we conducted MD simulations to evaluate the stability and dynamics of interactions between silybin, the most active component of silymarin, and the identified molecular targets, VEGFA and SRC.

The Root Mean Square Deviation (RMSD) analysis for the VEGFA–silybin and VEGFA-PGG complexes over a 100 ns molecular dynamics simulation, as shown in Figure 9A, offers key insights into the conformational stability of these complexes. The VEGFA–silybin complex exhibits initial RMSD values around 0.07 nm, which increase and stabilize at approximately 0.15 nm after 20 ns. This stabilization, with minor fluctuations, suggests that silybin forms a consistently stable complex with VEGFA throughout the simulation. Comparatively, the VEGFA-PGG complex starts with slightly lower initial RMSD values (~0.05 nm) and stabilizes around 0.12 nm after 20 ns, showing slightly less fluctuation overall. While the VEGFA-PGG complex demonstrates marginally lower RMSD values, the VEGFA–silybin complex remains stable, indicating that silybin is capable of forming robust and enduring interactions with VEGFA.

The Root Mean Square Fluctuation (RMSF) analysis, depicted in Figure 9B, highlights the flexibility of VEGFA residues when bound to silybin or PGG. The VEGFA–silybin complex displays slightly higher RMSF values for certain residue regions, such as residues 50, 120, and 180, with peak fluctuations reaching approximately 0.3 nm. This indicates that silybin introduces moderate flexibility in specific regions of the VEGFA structure. In contrast, the VEGFA-PGG complex exhibits lower RMSF values (ranging from 0.1 to 0.2 nm) across most residues, suggesting reduced flexibility.

The Solvent Accessible Surface Area (SASA) analysis, illustrated in Figure 9C, provides insights into the exposure of VEGFA’s surface to the solvent in the presence of the ligands. The VEGFA–silybin complex exhibits higher SASA values, fluctuating between 180 and 190 nm^2^ over the simulation period, indicating that silybin maintains VEGFA in a more open conformation. This could enhance the accessibility of certain binding sites, potentially favoring interactions with other biomolecules or cofactors. In comparison, the VEGFA-PGG complex shows lower SASA values (~170–180 nm^2^), reflecting tighter packing and reduced solvent exposure.

The radius of gyration (Rg) analysis, shown in Figure 9D, sheds light on the structural compactness of the VEGFA–silybin and VEGFA-PGG complexes. The VEGFA–silybin complex exhibits slightly higher Rg values (2.06–2.09 nm) with moderate fluctuations, suggesting a less compact but flexible structure. This contrasts with the VEGFA-PGG complex, which maintains lower and more stable Rg values (~2.05–2.07 nm).

The hydrogen bond (H-bond) analysis, illustrated in Figure 9E, reveals the dynamic interactions between VEGFA and the two ligands, silybin and PGG, during the 100 ns molecular dynamics simulation. Both VEGFA–silybin and VEGFA-PGG complexes demonstrate the potential to form up to eight hydrogen bonds throughout the simulation, highlighting their strong binding capacity. However, distinct patterns emerge in the frequency and stability of these interactions. The VEGFA–silybin complex exhibits a dynamic profile, with the number of H-bonds fluctuating between two and eight during the simulation. This variability suggests that silybin maintains strong hydrogen bonding interactions with VEGFA while allowing flexibility in the binding interface. In comparison, the VEGFA-PGG complex shows a more consistent H-bond profile, with the number of hydrogen bonds also fluctuating between two and eight but remaining closer to the upper range for longer durations. This indicates that PGG forms more stable hydrogen bonding interactions with VEGFA, leading to a more rigid and compact binding mode.

The RMSD analysis of the SRC-MC1 and SRC–silybin complexes over a 100 ns molecular dynamics simulation reveals important insights into their stability and conformational behavior (Figure 10A). Both complexes begin with RMSD values around 0.205 nm, indicating an initial state of stability. Throughout the simulation, the SRC-MC1 complex maintains relatively stable RMSD values, fluctuating slightly between 0.205 and 0.21 nm, demonstrating that the co-crystallized inhibitor MC1 ensures a stable conformation of the SRC protein. On the other hand, the SRC–silybin complex shows slightly higher fluctuations in RMSD, ranging from 0.205 to 0.215 nm. Despite these fluctuations, the RMSD values for SRC–silybin stabilize around 0.21 nm towards the end of the simulation, indicating that silybin binds stably to the SRC protein, albeit with slightly more conformational flexibility compared to MC1. Overall, both complexes exhibit stability, with the SRC-MC1 complex showing marginally better stability and lower RMSD fluctuations, while the SRC–silybin complex maintains structural integrity with minor conformational changes. This suggests that silybin is a viable ligand for SRC, capable of maintaining the protein’s structural integrity during binding.

The Root Mean Square Fluctuation (RMSF) plot provides insights into the flexibility of individual residues within the SRC-MC1 and SRC–silybin complexes (Figure 10B). For the SRC-MC1 complex, the RMSF values are generally lower, averaging around 0.06 ± 0.03 nm, indicating that most residues exhibit low flexibility. This suggests a stable interaction between the SRC protein and the MC1 inhibitor. The residues maintain their positions with minimal deviations, reflecting the rigidity of the complex. In comparison, the SRC–silybin complex shows slightly higher RMSF values, averaging 0.07 ± 0.03 nm. This indicates that certain residues exhibit more flexibility in this complex. The plot demonstrates peaks at specific residue positions, suggesting areas where the protein experiences more significant fluctuations. Despite these variations, the overall flexibility remains within a stable range, highlighting that silybin maintains a strong interaction with SRC while allowing for a bit more conformational freedom compared to MC1. The RMSF plot reveals specific regions within the protein that are more flexible. For both complexes, there are noticeable peaks around residues 50, 150, and 250, which could correspond to flexible segments within the SRC structure. The highest peak is observed near residue 270, indicating significant flexibility at this position, which could be part of a terminal or loop region that is inherently more dynamic.

The Solvent Accessible Surface Area (SASA) plot provides insights into the surface exposure of the SRC protein when complexed with either MC1 or silybin over the course of the molecular dynamics simulation (Figure 10C). The SASA values for the SRC-MC1 complex fluctuate between 175 nm^2^ and 180 nm^2^, with an average of 178.86 ± 1.80 nm^2^. This indicates that the SRC-MC1 complex maintains a relatively stable surface area exposed to the solvent, reflecting a consistent interaction between the protein and the inhibitor. The SASA values exhibit minor fluctuations, suggesting that the complex undergoes limited conformational changes and maintains a stable structure throughout the simulation. In contrast, the SRC–silybin complex shows SASA values that range between 175 nm^2^ and 185 nm^2^, with an average of 179.82 ± 1.66 nm^2^. This slightly higher average SASA indicates that the SRC–silybin complex has more surface area exposed to the solvent compared to the SRC-MC1 complex. The increased fluctuations in the SASA values for the SRC–silybin complex suggest that it experiences more significant conformational changes during the simulation. This could be due to the slightly more flexible interaction between SRC and silybin, allowing for more dynamic rearrangements of the protein surface.

The radius of gyration (Rg) plot, as shown in Figure 10D, measures the compactness of the SRC protein when complexed with MC1 and silybin over the course of the 100 ns molecular dynamics simulation. The Rg values for the SRC-MC1 complex fluctuate within a narrow range, generally between 2.06 nm and 2.09 nm. This consistent range indicates that the SRC-MC1 complex maintains a relatively stable and compact structure throughout the simulation. The stable Rg values suggest that the MC1 inhibitor helps maintain the structural integrity and compactness of the SRC protein. In contrast, the SRC–silybin complex exhibits slightly higher Rg values, fluctuating between 2.07 nm and 2.12 nm. The higher and more fluctuating Rg values compared to the SRC-MC1 complex indicate that the SRC–silybin complex experiences more significant conformational changes. However, these fluctuations stabilize around 2.10 nm towards the end of the simulation, suggesting that while silybin allows for more conformational freedom, it ultimately maintains a stable interaction with the SRC protein.

The number of H-bonds formed between the SRC protein and its ligands, MC1 and silybin, is a crucial determinant of the stability and strength of these complexes. The H-bonds graph (Figure 10E) illustrates the dynamics of these interactions over the 100 ns molecular dynamics simulation. For the SRC-MC1 complex, the number of hydrogen bonds fluctuates between 1 and 9, with an average of around 5-6 hydrogen bonds throughout the simulation. This relatively high and consistent number of hydrogen bonds indicates a strong and stable interaction between the SRC protein and the MC1 inhibitor. In comparison, the SRC–silybin complex exhibits fewer hydrogen bonds, fluctuating between 1 and 6, with an average of around 3-4 hydrogen bonds. This lower number of hydrogen bonds suggests that the interaction between SRC and silybin is slightly weaker than that of the SRC-MC1 complex. However, the hydrogen bond formation remains relatively stable throughout the simulation, supporting the notion that silybin maintains a significant interaction with the SRC protein.

The interaction of silybin with SRC, as revealed by the molecular dynamics simulation, shows that silybin binds stably to the SRC protein. The RMSD, RMSF, SASA, Rg, and hydrogen bond analyses collectively indicate that while the SRC–silybin complex exhibits slightly more flexibility and conformational changes, it maintains its structural integrity and significant interactions throughout the simulation. These findings suggest that silybin is a viable ligand for SRC and is capable of maintaining the protein’s structural integrity during binding.

## 5. Conclusions

This study employed an integrated in silico approach to investigate the therapeutic potential of silymarin in hepatocellular carcinoma (HCC). We identified 136 potential target genes for silymarin and 11,414 targets associated with HCC with 102 common targets, suggesting a possible overlap and therapeutic relevance. Network analysis highlighted VEGFA and SRC as potential key targets, with functional annotation and KEGG enrichment analyses indicating that silymarin may modulate multiple pathways, including the VEGF signaling pathway and proteoglycans in cancer. Molecular docking studies showed that silybin, isosilybin, and silychristin exhibited strong binding affinities for VEGFA and SRC, comparable to known inhibitors. Molecular dynamics simulations suggested the stability of these interactions, particularly for silybin. These findings provide a theoretical basis for the potential therapeutic role of silymarin in HCC. However, given the computational nature of this study, further experimental and clinical investigations are essential to validate these results and assess the practical applicability of silymarin as a multi-target therapeutic agent against HCC.

## Figures and Tables

**Table 1:** Docking results of silymarin active components and the reference inhibitor PGG with the highest degree hub target VEGFA (1VPF).

	Compounds	Binding Energy (Kcal/mol)	Hydrogen Interactions (Distance Å)	Number of Hydrogen Bonds	Hydrophobic Interactions	Electrostatic Interactions
**Silymarin active components**	Silybin	−7.7234	Cys61 (3.07), Gly59 (2.14), Gly58 (3.06), Leu32 (2.70), Leu32 (2.26), Leu32 (2.49), Asp34 (2.45), Asp34 (2.43), Asp63 (3.00), Leu66 (2.07)	10	Cys60, Cys68	Glu64
	Isosilybin	−7.6397	Cys61 (2.92), Glu64 (1.79), Asn62 (2.39), Asn62 (2.87), Gly58 (2.40), Gly58 (2.70), Gly59 (2.56), Asp63 (2.91), Ser63 (2.87)	09	Cys60, Cys68, Phe36	Anp34
	Silychristin	−6.7097	Glu64 (2.14), Gly59 (2.72), Gly59 (2.60), Asp34 (2.28)	04	Phe36, Ile46, Cys60	Glu64
**Reference inhibitor**	1,2,3,4,6-penta-O-galloyl-beta-D-glucose (PGG)	−8.0997	Cys68 (3.52), Cys61 (3.12), Asp34 (2.94), Gln37 (3.01), The31 (3.13), Ser50 (3.52), Val33 (2.75), Gly58 (2.41), Cys57 (2.84), Glu67 (3.07)	10	Pro70, Cys57, Cys61, Cys60	Glu64

**Table 2:** Docking results of silymarin active components with the highest degree hub target SRC (3U51).

	Compounds	Binding Energy (Kcal/mol)	Hydrogen Interactions (Distance Å)	Number of Hydrogen Bonds	Hydrophobic Interactions	Electrostatic Interactions
**Silymarin active components**	Silybin	−8.7466	Cys277 (2.93), Asp404 (2.25), Gln275 (2.92), Gln275 (2.57), Gln275 (2.49), Gly274 (2.55), Gly274 (2.87), Gly406 (2.36), Asp386 (2.45)	09	Phe278, Gln275, Val281, Leu393	Cys277, Asp404
	Isosilybin	−8.7382	Ala403 (2.68), Asp386 (1.87), Asp404 (2.80), Asp404 (2.63), Glu280 (2.60)	05	Val281, Gln275, Cys277	Lys295, Met314, Asp404
	Silychristin	−8.6096	Glu280 (2.77), Gly279 (2.21), Gly279 (2.82), Leu273 (2.77), Gly344 (1.94), Asp348 (2.73), Asp348 (2.54)	07	Leu393, Val281, Leu273	Asp404, Cys177
**Co-crystallized inhibitor**	Macrocyclic inhibitor MC1	−10.6949	Ala390 (1.88), Ser345 (2.26), Ser345 (3.02), Asp348 (2.81), Asp404 (1.91), Asp404 (1.88), Asp404 (1.81), Phe278 (2.62), Gly279 (2.32), Gly279 (2.34), Gly276 (1.69), Glu280 (3.83), Lys295 (1.86), Met341 (2.91), Met341 (2.80), Glu339 (1.88), Gln275 (2.69)	17	Ile336, Gly279, Cys277	Asp404, Asp348

**Table 3:** The average values of Rg, SASA, RMSD, and RMSF and the number of H-bonds for the studied complexes.

Complex	Average RMSD (nm)	Average RMSF (nm)	Average Rg (nm)	Average SASA (nm^2^)	Max Number of H-Bond
**VEGFA-PGG**	0.12 ± 0.01	0.05 ± 0.04	2.05 ± 0.02	179.42 ± 3.07	7
**VEGFA–silybin**	0.14 ± 0.01	0.07 ± 0.03	2.07 ± 0.07	187.85 ± 3.10	7
**SRC-MC1**	0.20 ± 0.01	0.06 ± 0.03	2.08 ± 0.06	178.86 ± 1.80	6
**SRC–silybin**	0.21 ± 0.01	0.07 ± 0.03	2.10 ± 0.07	179.82 ± 1.66	9

**Table 4:** Results showing the electrostatic, van der Waals, SASA, polar salvation, and binding energy in kJ mol^−1^ for the studied complexes.

Protein–Ligand Complex	Van der Waals Energy	Electrostatic Energy	Polar Salvation	SASA Energy	Total Energy (kJ mol^−1^)
**VEGFA-PGG**	−102.16 ± 9.05	−4.25 ± 2.05	19.36 ± 8.57	−9.21± 2.18	−96.26 ± 11.32
**VEGFA–silybin**	−89.47 ± 11.25	−5.02 ± 0.35	22.18 ± 13.15	−5.10 ± 0.42	−77.41 ± 12.95
**SRC-MC1**	−200.78 ± 7.95	−17.55 ± 1.92	45.60 ± 6.75	−12.02 ± 0.65	−184.75 ± 13.70
**SRC–silybin**	−148.32 ± 10.68	−22.05 ± 2.65	38.50 ± 8.12	−11.75 ± 1.59	−143.62 ± 12.88

## Data Availability

The original contributions presented in the study are included in the article/Supplementary Materials. Further inquiries can be directed to the corresponding authors.

## Supplementary Materials

The following supporting information can be downloaded at https://www.mdpi.com/article/10.3390/metabo15010053/s1.
