# Supplementary material for: Silymarin as a Therapeutic Agent for Hepatocellular Carcinoma: A Multi-Approach Computational Study
Source: Metabolites. 2025 Jan 15;15(1):53. doi: 10.3390/metabo15010053 (PMC11767256; doi:10.3390/metabo15010053)
Supplement: Supplementary file 1 [file metabolites-15-00053-s001.zip › metabolites-3357568-supplementary.pdf]

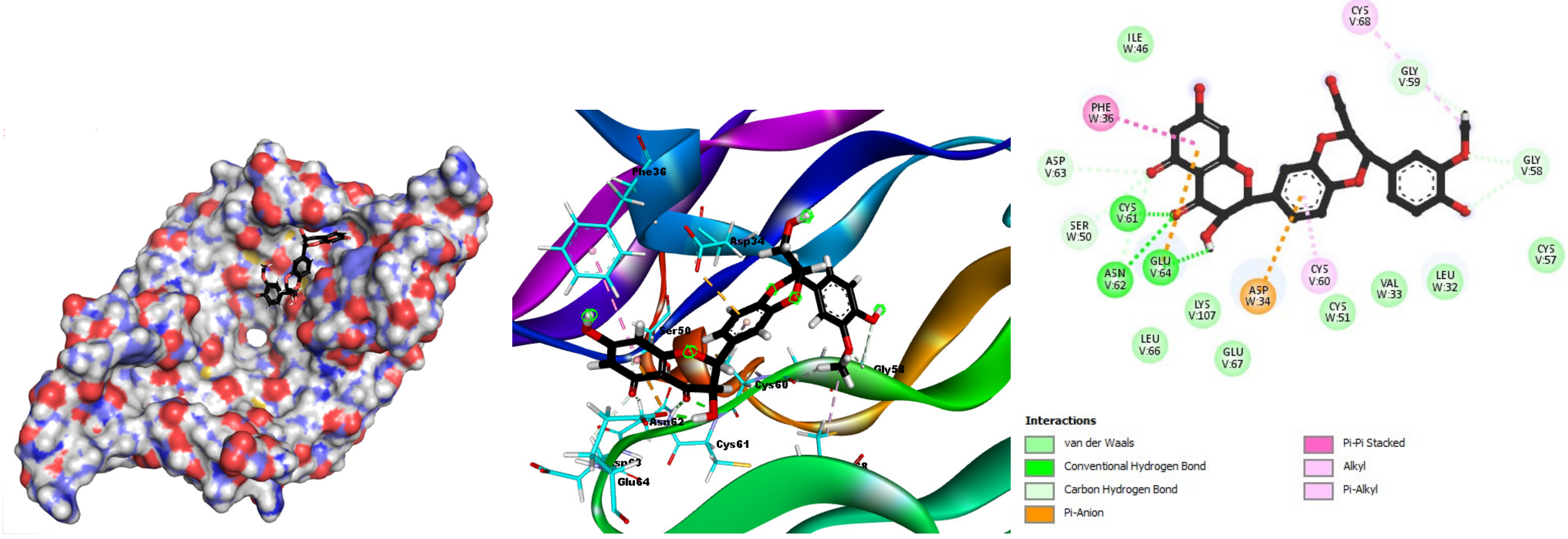

Isosilybin

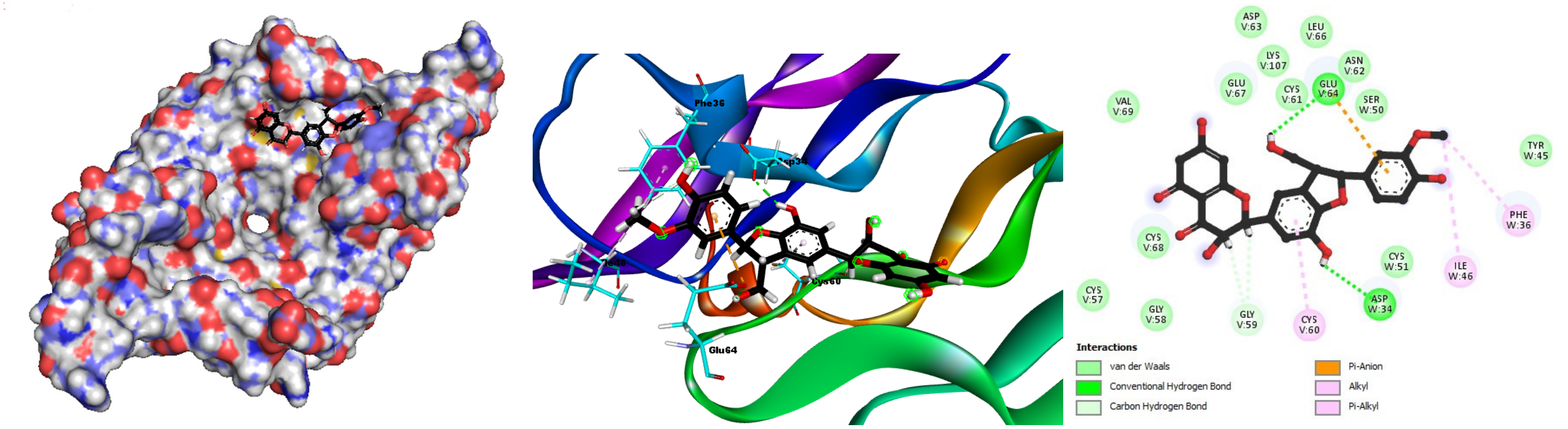

Silychristin

Supplemental Figure S1. Molecular docking 2D and 3D diagrams of Isosilybin and Silychristin with the highest degree hub target VEGFA (1VPF).

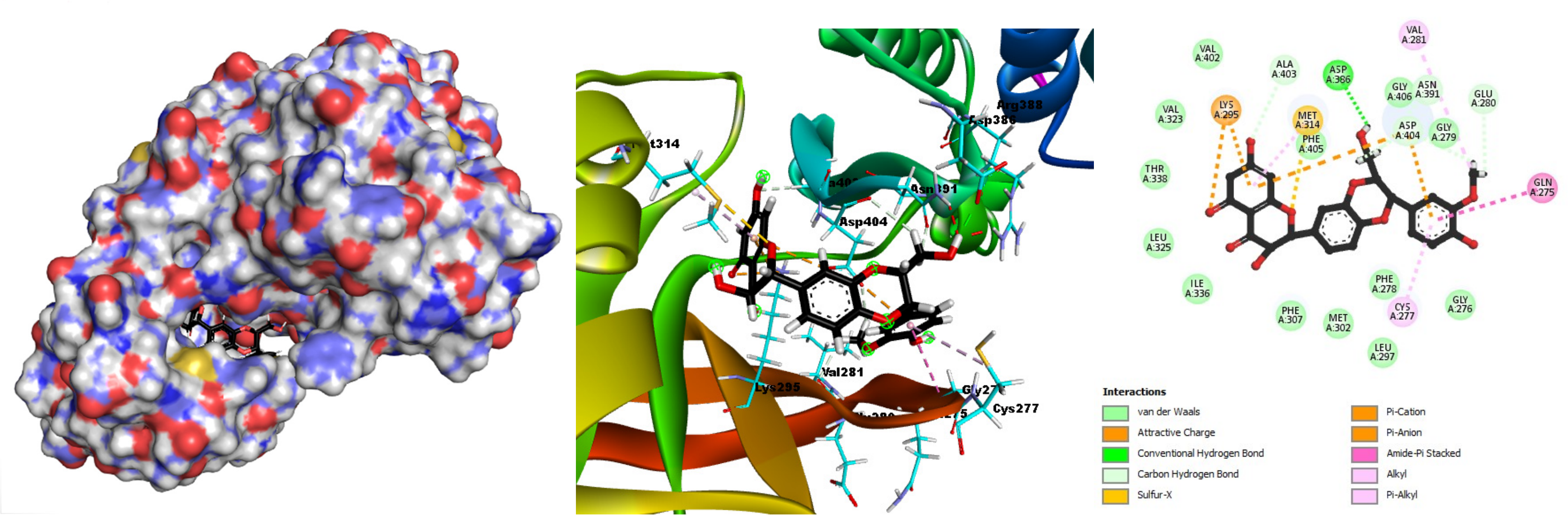

Isosilybin

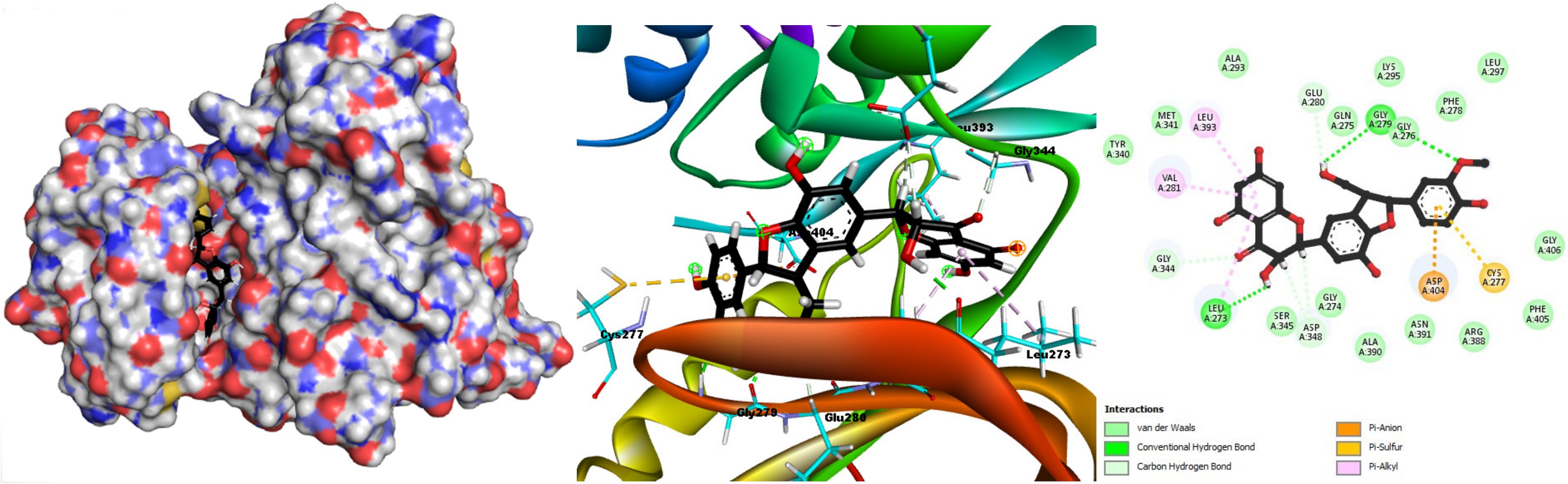

Silychristin

Supplemental Figure S2. Molecular docking 2D and 3D diagrams of Isosilybin and Silychristin with the highest degree hub target SRC (3U51).
